# Vaginal Bleeding Due to Iatrogenic Uterine Perforation – A Case Report

**DOI:** 10.21980/J83643

**Published:** 2024-04-30

**Authors:** John Costumbrado, Leah Snyder, Sassan Ghassemzadeh, Daniel Ng

**Affiliations:** * University of California, Riverside, School of Medicine, Riverside, CA; ^ Riverside Community Hospital, Department of Emergency Medicine, Riverside, CA

## Abstract

Uterine perforation is a rare but potentially life-threatening complication of gynecologic procedures. Serious complications include hemorrhage, infection, and injury to surrounding organ systems (eg, gastrointestinal, urological, vascular, etc.). Risk factors include advanced maternal age, prior gynecologic surgeries, and other anatomical features that impact the difficulty of accessing the uterine cavity. In this case report, we discuss a patient who presented to the emergency department (ED) with diffuse abdominal pain and vaginal bleeding that occurred after an elective dilation and curettage (D&C) for a termination of pregnancy. The diagnosis was suspected clinically and confirmed by imaging including ultrasound (US) and computed tomography (CT) of the abdomen and pelvis. The patient was managed operatively with a multidisciplinary approach including Gynecology, General Surgery, and Urology. The patient was stabilized and eventually discharged. Uterine perforation should be included in the differential for patients with a history of recent gynecologic instrumentation presenting with abdominal pain and vaginal bleeding. The stabilization of these patients requires aggressive volume resuscitation, controlling the source of bleeding, and emergent surgical consultation.

**Topics:**

Gynecology, vaginal bleeding, ultrasound, computed tomography.

[Fig f1-jetem-9-2-v6] [Fig f2-jetem-9-2-v6]

**Figure f1-jetem-9-2-v6:**
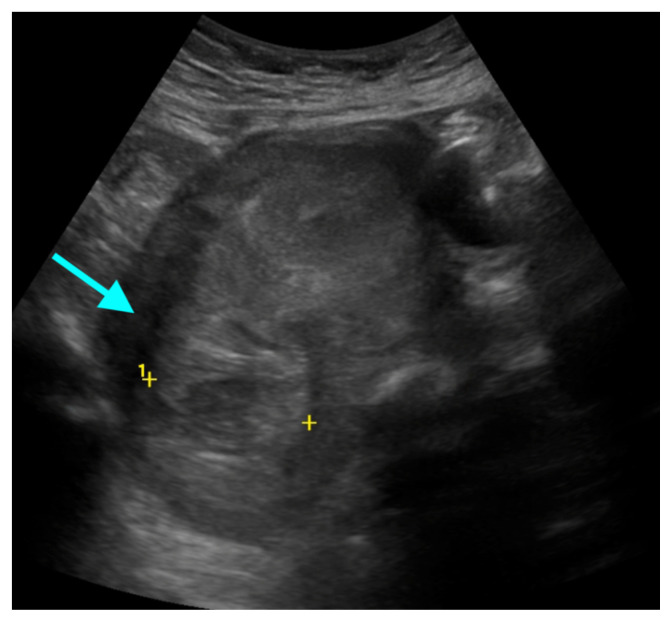


**Figure f2-jetem-9-2-v6:**
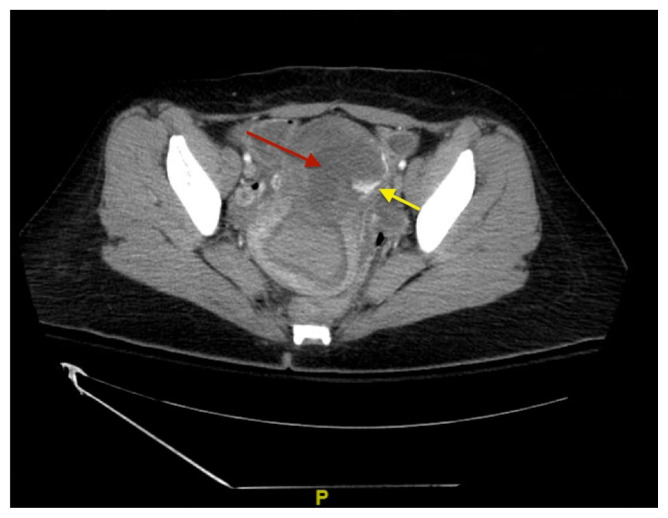


## Brief introduction

Uterine perforation is a rare complication occurring in less than 0.3% in the first and second-trimester procedural pregnancy terminations.^1^ It occurs most commonly during mechanical dilation of the cervix or insertion of sharp instruments into the uterus.^2^ Risk factors include the level of surgical expertise, advanced maternal age, greater parity, increased gestational age, abnormal uterine and/or cervical anatomy, cervical stenosis, and history of prior Cesarean section.^3^,^4^ Uterine rupture can cause significant morbidity and mortality. Significant, life-threatening hemorrhage requiring resuscitation, damage to surrounding genitourinary structures, and need for hysterectomy in the event of irreparable damage are the most common complications.^5^

## Presenting concerns and clinical findings

A 35-year-old female (gravida 4, para 3 via 3 Cesarean sections) at approximately nine weeks of gestation presented to the ED for abdominal pain and severe vaginal bleeding that developed during an outpatient elective pregnancy termination with D&C. During the procedure, the patient became hypotensive with significant vaginal bleeding noted, estimated at approximately 1 L. Patient was found to have systolic blood pressure in the 70’s in the field, and was administered 1.5 L of intravenous (IV) fluids by paramedics. Upon arrival, she was immediately placed in a resuscitation room with orders for 1 gm tranexamic acid (TXA) and 2 units of packed red blood cells (pRBC). In the ED, her initial blood pressure was 113/56 mm Hg with a heart rate of 68 bpm. The rest of vitals were within normal limits. The patient was complaining of diffuse abdominal pain for which she was given 50 mcg of IV fentanyl. The differential diagnosis included uterine perforation, bowel perforation, and vascular injury. A bedside focused assessment with sonography in trauma (FAST) exam was performed and did not show evidence of free fluid; however, there was an abnormal intrauterine mass suspected to represent a hematoma. In the setting of her recent procedure and presentation, gynecology was emergently consulted to evaluate the patient. Her pelvic exam was positive for blood in the vaginal vault, but with no active hemorrhage. Intravaginal packing was considered; however, it was thought that the suspected hematoma was tamponading the hemorrhage. A venous blood gas estimated the patient’s hemoglobin at 9.4 g/dL with an unknown baseline value. Given the patient’s hemodynamic stability, Gynecology recommended CT of the abdomen and pelvis to assess the source of the bleeding. Computed tomography was completed and demonstrated findings concerning for uterine perforation.

## Significant findings

The bedside transabdominal US of the pelvis showed a heterogeneous mixture of hypoechoic and hyperechoic endometrial thickening extending to the lower uterine segment (blue arrow), which was thought to represent active hemorrhage. Computed tomography of the abdomen and pelvis showed evidence of a large amount of endometrial hyperdensity (red arrow) suggestive of hemorrhagic contents within a grossly enlarged uterus. There was relative decreased enhancement of the uterine body and fundus, concerning for devascularization. There was also active extravasation along the left lateral uterus (yellow arrow).

## Patient course

In the ED, the patient was found to have evidence of uterine perforation on imaging. Antibiotics, 1 gm of IV ceftriaxone and 500 mg of IV metronidazole, were administered to broadly cover organisms associated with perforation. Her initial hemoglobin of 9.4 g/dL on point-of-care testing was similar to the value of 9.6 g/dL on formal hematology results. Given the extent of bleeding, Gynecology planned to take the patient to the operating room (OR) for diagnostic laparotomy. Her hemoglobin dropped to 7.4 g/dL prior to going to the OR and 1 unit pRBC was transfused. In the OR, the patient was found to have a large, left-sided broad ligament hematoma without evidence of active bleeding. While in the OR, the patient developed profuse vaginal bleeding and the decision was made to emergently proceed with a total abdominal hysterectomy and bilateral salpingectomy for hemorrhagic control. Intraoperatively, she received 2 more units of pRBCs and both General Surgery and Urology were consulted to assess for bowel and ureteral injuries, respectively. There was no evidence of other injuries. The procedure was successful and her postoperative course was uncomplicated. On postoperative day 3, the patient’s pain was well-controlled, she was tolerating oral intake, and ambulating. Her last hemoglobin was 9.1 g/dL and she was discharged in stable condition.

## Discussion

In this case, while the obstetric D&C alone increased the patient’s risk for perforation, the patient had other risk factors including decreased myometrial strength (multiparity and advanced maternal age) and possible scarring from previous Cesarean sections.^6^,^7^ In the ED, stabilizing a patient with significant hemorrhage is essential. A strength of this case report for learners is the intersection between acute trauma management and gynecological emergencies. The ED clinicians were prepared to transfuse blood and administer TXA given the prehospital report of the patient’s history and hemodynamics. The collaborative efforts of the ED team’s and prehospitalist’s interventions facilitated radiographic evaluation. The combination of US and CT imaging confirmed the diagnosis of uterine rupture, leading gynecology to determine the necessity for operative management. The patient was stabilized after operative management, and the remainder of her hospital stay was uncomplicated.

Management of uterine perforation begins with the evaluation of hemodynamic stability and resuscitation. Large bore IV access and early pRBC administration is prioritized. Tranexamic acid should also be considered and discussed with Gynecology. Tranexamic acid is FDA approved for significant menstrual bleeding but often used off-label for other sources of massive hemorrhage. In a randomized controlled trial (RCT) of nearly 15,000 women with massive post-partum hemorrhage, there was a significant decrease in mortality risk when TXA was administered with a RR of 0.41.^8^ Obtaining imaging to evaluate for uterine rupture and damage to surrounding structures depends on the stability of the patient. If damage to the genitourinary system is suspected on exam or imaging, urological services should be consulted. Definitive treatment revolves around either conservative management with repair of the rupture or hysterectomy. The decision between these two treatments depends on the extent and location of the rupture, the woman’s reproductive considerations, and damage to associated structures. Because the incidence of rupture is low, literature to compare the morbidity and mortality of these management strategies is limited, and thus the ultimate management of these patients is largely based on the surgeon’s discretion.^8^ A limitation of this case report is that there is no information available regarding the long-term outcomes for the patient.

Regarding imaging, US is the study of choice for suspected uterine perforation due to accessibility, mobility to perform the study in the ED, as well as cost and lack of ionizing radiation.^6^ The most common US features of uterine perforation include heterogeneous intrauterine content (which was present in this case), hemoperitoneum, and pneumoperitoneum.^9^ US imaging has a sensitivity of 93% and specificity of 64% for detecting pneumoperitoneum.^10^ If US is negative or inconclusive for pneumoperitoneum, CT can be an adjunct modality in detecting evidence of perforation in stabilized patients.^6^ Magnetic resonance imaging (MRI) has a limited role in emergent scenarios; however, MRI has a superior soft-tissue resolution and can improve the identification of uterine perforation with associated complications, such as secondary abscess formation in stable patients.^11^

Uterine perforation should be a leading differential for patients with abdominal pain, vaginal bleeding, and recent gynecologic procedures such as D&C. Perforation is a severe and life-threatening diagnosis due to significant bleeding and other complications such as infection that can progress to sepsis. Stabilizing hemorrhaging patients includes establishing large-bore IV access, initiating prompt volume resuscitation with a low-threshold for emergent blood transfusion, and additional adjuncts for hemorrhage control. Early administration of broad-spectrum antibiotics against both aerobic and anaerobic bacteria is essential to reduce morbidity and mortality associated with intra-abdominal and pelvic infections that can quickly progress to sepsis. Emergent gynecology consultation is critical and patients with suspected uterine perforation may be managed emergently or operatively in cases of severe uterine bleeding or suspected injuries to other organs.

## Supplementary Information








