# Supplementary figures and images for: Vaginal Bleeding Due to Iatrogenic Uterine Perforation – A Case Report
Source: J Educ Teach Emerg Med. 2024 Apr 30;9(2):V6–9. doi: 10.21980/J83643 (PMC11068317; doi:10.21980/J83643)

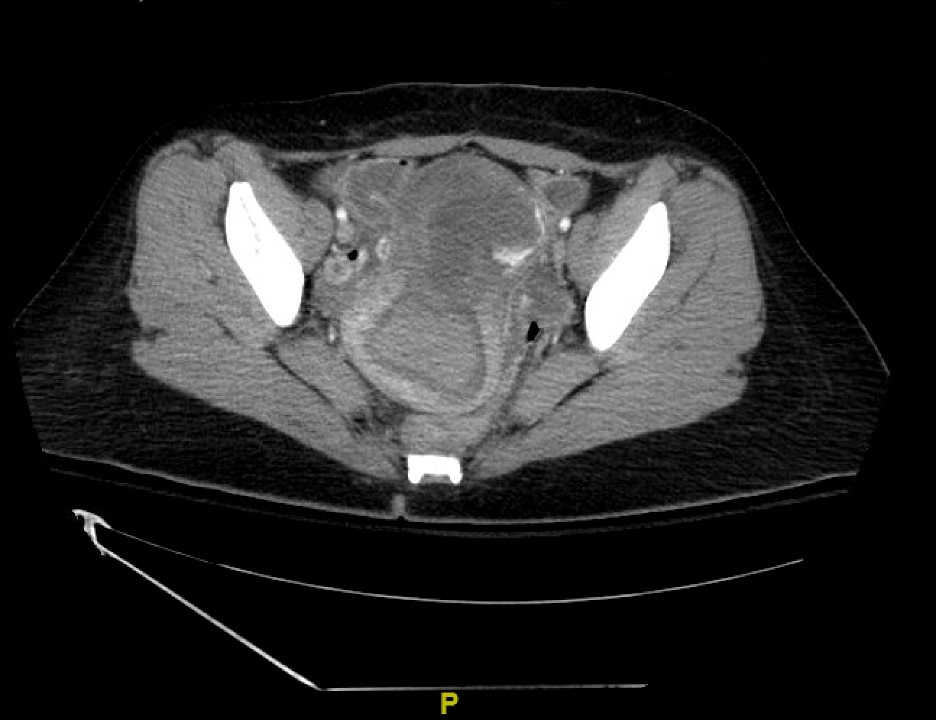

Supplement: Supplementary file 1 [file jetem-9-2-V6-supp1.jpg]

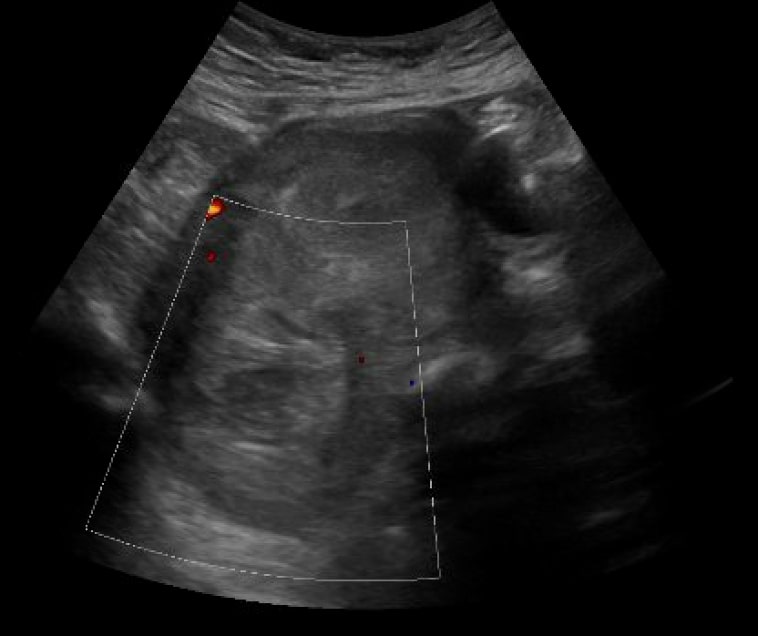

Supplement: Supplementary file 2 [file jetem-9-2-V6-supp2.jpg]

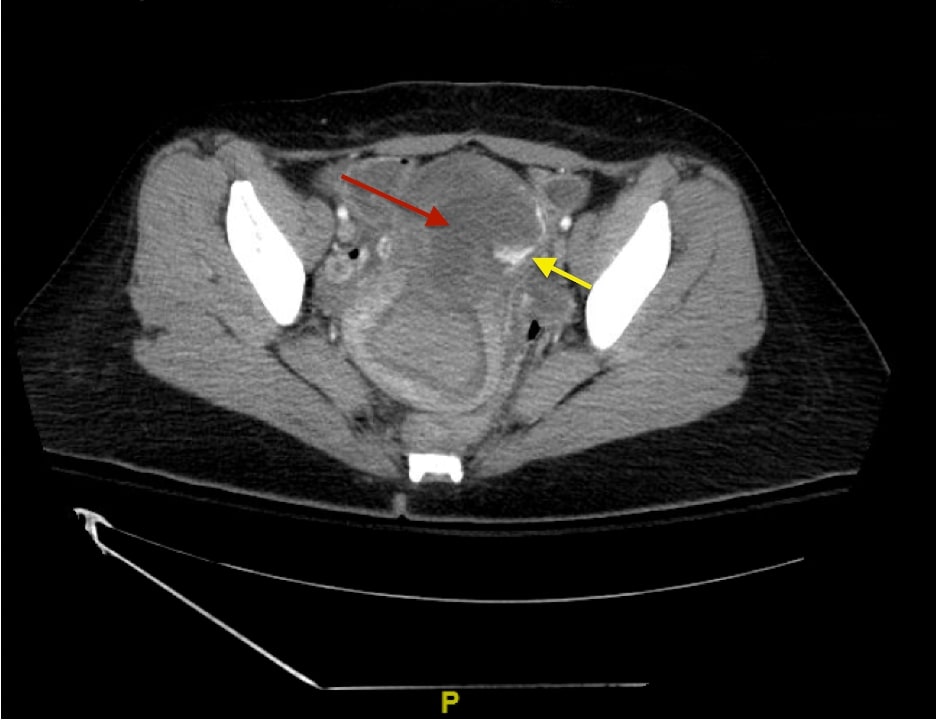

Supplement: Supplementary file 3 [file jetem-9-2-V6-supp3.jpg]

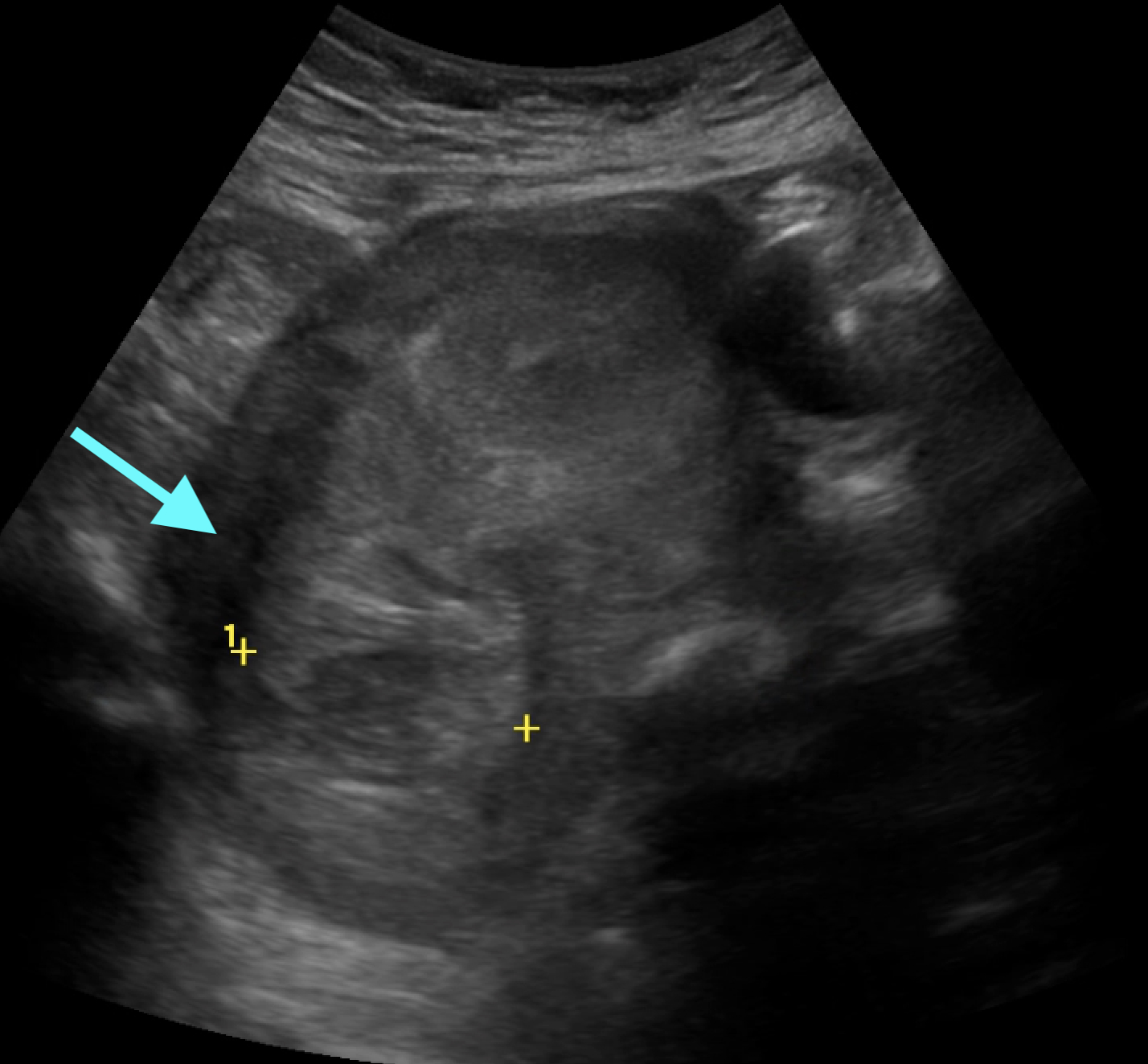

Supplement: Supplementary file 4 [file jetem-9-2-V6-supp4.jpg]
